# Computation of the smooth max-mutual information via semidefinite programming

**DOI:** 10.1007/s11128-026-05101-8

**Published:** 2026-02-25

**Authors:** Christopher Popp, Tobias C. Sutter, Beatrix C. Hiesmayr

**Affiliations:** Faculty of Physics, University of Vienna, Währinger Straße 17, 1090 Vienna, Austria

**Keywords:** Quantum information, Semidefinite programming, Smooth max-mutual information, Smooth max-relative entropy

## Abstract

We present an iterative algorithm based on semidefinite programming (SDP) for computing the quantum smooth max-mutual information $I^\varepsilon _{\max }(\rho _{AB})$ of bipartite quantum states in any dimension. The algorithm is accurate if a rank condition for marginal states within the smoothing environment is satisfied and provides an upper bound otherwise. Central to our method is a novel SDP, for which we establish primal and dual formulations and prove strong duality. With the direct application of bounding the one-shot distillable key of a quantum state, this contribution extends SDP-based techniques in quantum information theory. Thereby it improves the capabilities to compute or estimate information measures with application to various quantum information processing tasks.

## Introduction

Quantum information theory has extended our understanding of information processing, communication, and cryptography by leveraging the principles of quantum mechanics [1]. Central to this field are entropic quantities that quantify correlations, uncertainties, and operational capabilities of quantum systems (cf. [2] for a comprehensive overview). In particular, considering small deviations from the quantum states at hand, smoothed versions of entropic and information measures, such as the smooth max-mutual information $I^\varepsilon _{max}(\rho _{AB})$ [3, 4] for bipartite quantum states $\rho _{AB}$ are essential for characterizing one-shot operational tasks (cf. Ref. [5]) in noisy environments with finite resources.

The smooth max-mutual information captures the correlation between subsystems *A* and *B*, accounting for small errors due to noise or approximations due to limited resources. This quantity is particularly relevant in cryptographic settings, where it can be combined with other measures like the hypothesis testing mutual information [6] to bound the one-shot distillable key [7]. This provides practical estimates for secure key rates achievable from quantum states [2]. However, computing $I^\varepsilon _{max}$ exactly remains a computationally challenging optimization problem. Existing methods in quantum information theory have increasingly relied on semidefinite programming (SDP) to compute, approximate, or bound entropic quantities efficiently [8–12]. For instance, SDP formulations have been successfully applied to compute the hypothesis testing mutual information and conditional smooth min- and max-entropies [13].

Despite these advances, an SDP-based approach for the smooth max-mutual information is lacking. In Ref. [13], the authors recently used a so-called mountain-climbing-type algorithm [14] to use SDPs iteratively to calculate the smooth min-entropy, an information quantity not allowing a direct formulation as an SDP. In this work, we employ a similar approach by introducing an SDP-based iterative algorithm for computing $I^\varepsilon _{max}(\rho _{AB})$. Crucially, the quantity considered in Ref. [13] and $I^\varepsilon _{max}$ differ in their smoothing structure, requiring an alternative approach, including a novel SDP at the core of the algorithm. We characterize this SDP by its prime-dual formulations, show strong duality, and prove that the iterative algorithm computes the smooth max-mutual information accurately, assuming a rank constraint regarding the marginal states in the smoothing neighborhood.

Section 2 establishes the notation and the information quantities and SDP methods applied in this work. In Sect. 3, we present an SDP-based algorithm to compute the smooth max-mutual information. We characterize the underlying SDP by its primal and dual form and prove the accuracy of the algorithm under the assumption of a rank constraint. Finally, we conclude our results in Sect. 4.

## Background

### Notation

Consider the bipartite Hilbert space $\mathcal {H}= \mathcal {H}_A \otimes \mathcal {H}_B$ of two parties, *A* and *B*. The set of linear operators on $\mathcal {H}$ is denoted by $L(\mathcal {H})$. The set of positive semidefinite operators $X\ge 0$ is denoted as $L_+(\mathcal {H})$ and the set of density operators, or quantum states, satisfying $\rho \in L_+(\mathcal {H})$ and ${{\,\textrm{Tr}\,}}(\rho )=1$ is written as $\mathcal {D}(\mathcal {H})$. The marginal states of a multipartite state are denoted as $\rho _{A/B}:={{\,\textrm{Tr}\,}}_{B/A}[\rho _{AB}]$. If *X* is positive definite, we denote it by $X > 0$. A superoperator $\Phi$ is a linear operator mapping $L(\mathcal {H}) \rightarrow L(\mathcal {H})$. $\Phi$ is hermiticity preserving, if $\Phi (X)$ is hermitian for every hermitian *X*. The adjoint superoperator $\Phi ^\dagger$ is defined by satisfying ${{\,\textrm{Tr}\,}}[\Phi (X)Y] = {{\,\textrm{Tr}\,}}[X\Phi ^\dagger (Y)]$ for all $X,Y \in L(\mathcal {H})$.

### The max-relative entropy and the smooth max-mutual information

The information measure analyzed in this work involves an optimization over an environment around the quantum state of interest, called smoothing. For that purpose, we use the so-called sine distance [15] based on the fidelity [16]:

#### Definition 1

*(Fidelity, sine distance)* The fidelity of two quantum states $\rho$ and $\sigma$ is defined as follows:

1 $$\begin{aligned} \mathcal {F}(\rho , \sigma ) := \left( {{\,\textrm{Tr}\,}}[\sqrt{\sqrt{\sigma } \rho \sqrt{\sigma }}]\right) ^2 \end{aligned}$$

The sine distance is defined as:

2 $$\begin{aligned} P(\rho ,\sigma ) := \sqrt{1-\mathcal {F}(\rho ,\sigma )} \end{aligned}$$

Using this distance measure, the smoothing environment is defined as a ball around the quantum state for which the information measure is to be evaluated.

#### Definition 2

*(Smoothing environment)* Using the sine distance, the smoothing environment $B^{\varepsilon }(\rho )$ around a state $\rho$ is defined as

3 $$\begin{aligned} B^\varepsilon (\rho ) := \left\{ \tilde{\rho } \in \mathcal {D}(\mathcal {H}): P(\rho ,\tilde{\rho }) \le \varepsilon \right\} = \left\{ \tilde{\rho } : \mathcal {F}(\rho , \tilde{\rho }) \ge 1-\varepsilon ^2 \right\} . \end{aligned}$$

The quantity of interest in this work is related to the max-relative entropy $D_{max}$ [4]. Here, we analyze a smoothed generalized mutual information $I^\varepsilon _{max}$ [3] based on $D_{max}$, which can be used for quantifying the capability of a quantum state to be used for the task of secret-key distillation (cf., e.g., [2, 7]).

#### Definition 3

*(Max-relative entropy, smooth max-mutual information)*

4 $$\begin{aligned} D_{max}(\rho ||\sigma )&:= {\left\{ \begin{array}{ll} \log _2 \left\| \sigma ^{-\frac{1}{2}} \rho \sigma ^{-\frac{1}{2}} \right\| _{\infty } ~~~\text { if supp}(\rho ) \subseteq \text {supp}(\sigma ) \\ + \infty ~~~~~~~~~~~~~~~~~~~~~~~~ \text { else} \end{array}\right. } \end{aligned}$$

5 $$\begin{aligned} I_{max}^\varepsilon (\rho _{AB})&:= \inf _{\tilde{\rho }_{AB} \in B^\varepsilon (\rho _{AB})} D_{max}(\tilde{\rho }_{AB} || \rho _A \otimes \tilde{\rho }_B) \end{aligned}$$

where $B^\varepsilon (\rho _{AB})$ is as in Definition 2, $|| \cdot ||_\infty$ is the spectral norm and $\tilde{\rho }_B:= {{\,\textrm{Tr}\,}}_A[\tilde{\rho }_{AB}]$.

### Semidefinite programming

Semidefinite programs are constrained optimization problems with positive semidefinite optimization variables that often occur in quantum information theory.

#### Definition 4

*(Semidefinite program)* Let $\Phi$ be a hermiticity preserving superoperator and *A* and *B* be hermitian operators. A semidefinite program (SDP) corresponds to the following two optimization problems over positive semidefinite operators *X*, *Y*.

The primal SDP:

6 $$\begin{aligned}&\text {maximize } {{\,\textrm{Tr}\,}}[AX] \nonumber \\&\text {subject to }~ \Phi (X) \le B, ~ X\ge 0. \end{aligned}$$

The dual SDP:

7 $$\begin{aligned}&\text {minimize } {{\,\textrm{Tr}\,}}[BY] \nonumber \\&\text {subject to }~ \Phi ^\dagger (Y) \ge A, ~ Y\ge 0, \end{aligned}$$

where $\Phi ^\dagger$ is the adjoint superoperator of $\Phi$.

A variable *X* satisfying the constraints of the primal SDP is called a feasible point. If *X* satisfies $\Phi (X)<B$ and $X > 0$, it is called a strictly feasible point. Similarly, *Y* is feasible if it satisfies the constraints of the dual SDP and strictly feasible if the constraints are strictly satisfied. An SDP is said to satisfy the strong duality property if the optimal values of (6) and (7) of the primal and dual problems are equal.

## Computing the smooth max-mutual information with an SDP-based algorithm

The max-relative entropy $D_{max}(\rho ||\sigma )$ of two quantum states $\rho$ and $\sigma$ can be characterized by the following optimization problem (cf. Ref. [2]):

8 $$\begin{aligned} D_{max}(\rho ||\sigma ) = \log _2 \inf _{\lambda \ge 0} \lbrace \lambda : \lambda \sigma - \rho \ge 0 \rbrace . \end{aligned}$$

The inner optimization can be formulated as an SDP in its dual form by setting $Y\equiv \lambda ,~A=\rho , ~B = 1, ~\Phi ^\dagger (Y)=Y\sigma$ in Definition 4. The same does not hold for the smooth max-mutual information $I^\varepsilon _{max}(\rho _{AB})$. This can be seen by writing $I_{max}^\varepsilon$ using the characterization for the max-relative entropy (8), implying

9 $$\begin{aligned} I_{max}^\varepsilon (\rho _{AB}) := \log _2 \left( \inf _{\tilde{\rho }_{AB} \in B^\varepsilon (\rho _{AB})} \inf _{\lambda \ge 0} \left\{ \lambda : \lambda \rho _A \otimes \tilde{\rho }_B - \tilde{\rho }_{AB} \ge 0 \right\} \right) := \log _2(\lambda _{min}). \end{aligned}$$

As the constraint involves a bilinear term in the variables $\tilde{\rho }_{AB}$ and $\lambda$, this cannot be directly solved by a SDP. We propose an iterative approach applicable for states $\rho _{AB}$ and $\varepsilon > 0$ for which $B^\varepsilon (\rho _{AB})$ contains only full-rank marginal states $\rho _A:= {{\,\textrm{Tr}\,}}_B[\rho _{AB}]$ and $\tilde{\rho }_B:={{\,\textrm{Tr}\,}}_A[\tilde{\rho }_{AB}]$. Alternately fixing one of the variables and solving an SDP for the other, this seesaw or mountain-climbing algorithm converges to the optimal solution after a finite amount of iterations [14], providing an upper bound approximating $I^{\varepsilon }_{max}(\rho _{AB})$. Define the *i*-th iteration of the procedure as follows:

### Algorithm 1

1. If $$i=1$$, choose $$\rho _{AB}^i = \rho _{AB}$$.
2. Solve the following SDP, with the solution defining
  10 $$\begin{aligned} \lambda ^i := \inf _{\lambda \ge 0} \left\{ \lambda : \lambda \rho _A \otimes \rho _B^i - \rho _{AB}^i \ge 0 \right\} . \end{aligned}$$
3. Solve the following SDP, with the solution defining
  11 $$\begin{aligned} \mu ^i := \sup _{\begin{array}{c} \mu \ge 0, \\ \tilde{\rho }_{AB}\in B^\varepsilon (\rho _{AB}) \end{array}} \left\{ \mu : \lambda ^i \rho _A \otimes \tilde{\rho }_B - \tilde{\rho }_{AB} - \mu \mathbbm {1} \ge 0 \right\} \end{aligned}$$
4. If $\mu ^i = 0$ stop and set $\lambda _{min} = \lambda ^i$. Else define $\rho _{AB}^{i+1}$ to be state $\tilde{\rho }_{AB} \in B^\varepsilon (\rho _{AB})$ maximizing the expression in step 3 and use it in step 2 for the next iteration.

Note that for all iterations, $B^\varepsilon (\rho _{AB})$ remains fixed around $\rho _{AB}$. We now characterize the SDP of (11) by its primal and dual form and show strong duality.

### Proposition 1

The primal and dual SDPs of

12 $$\begin{aligned} \sup _{\begin{array}{c} \mu \ge 0, \\ \tilde{\rho }_{AB}\in B^\varepsilon (\rho _{AB}) \end{array}} \left\{ \mu : \lambda ^i \rho _A \otimes \tilde{\rho }_B - \tilde{\rho }_{AB} - \mu \mathbbm {1} \ge 0 \right\} \end{aligned}$$

can be written in the following forms, for which strong duality holds.

13 $$\begin{aligned}&\mathrm {Primal:} \nonumber \\&\sup _{\begin{array}{c} \mu \ge 0, \\ \tilde{\rho }_{AB}\in \mathcal {D}(\mathcal {H}), \\ X \in L(\mathcal {H}) \end{array}} \left\{ \mu : \lambda \rho _A \otimes \tilde{\rho }_B - \tilde{\rho }_{AB} - \mu \mathbbm {1} \ge 0, ~\textrm{Re} \lbrace {{\,\textrm{Tr}\,}}[X]\rbrace \ge \sqrt{1-\varepsilon ^2}, ~\begin{pmatrix} \rho _{AB} &  X \\ X^\dagger &  \tilde{\rho }_{AB} \end{pmatrix} \ge 0 \right\} \end{aligned}$$

14 $$\begin{aligned}&\mathrm {Dual:} \nonumber \\&\inf _{\begin{array}{c} W, D \ge 0\\ \eta \in \mathbbm {R}, \nu \ge 0 \end{array}} \left\{ \eta - 2\nu \sqrt{1-\varepsilon ^2} + {{\,\textrm{Tr}\,}}[\rho D]: ~ {{\,\textrm{Tr}\,}}[W] \ge 1, ~ \begin{pmatrix} D &  \nu \mathbbm {1} \\ \nu \mathbbm {1} &  \eta \mathbbm {1}- (\lambda M -W)\end{pmatrix} \ge 0 \right\} . \end{aligned}$$

### Proof

First note that by (3), the $\varepsilon$-ball based on the sine distance can be characterized by the root fidelity, for which we can use the characterization $$\sqrt{\mathcal {F}}(\rho ,\tilde{\rho }) := \sqrt{\mathcal {F}(\rho ,\tilde{\rho })}= \sup _{X \in L(\mathcal {H})} \left\{ \textrm{Re} \lbrace {{\,\textrm{Tr}\,}}[X] \rbrace : \begin{pmatrix} \rho &  X \\ X^\dagger &  \tilde{\rho } \end{pmatrix} \ge 0 \right\} $$ [11] to write the optimization problem in (12) as:

15 $$\begin{aligned} \sup _{\begin{array}{c} \mu \ge 0, \\ \tilde{\rho }_{AB}\in \mathcal {D}(\mathcal {H}, \\ X \in L(\mathcal {H}) \end{array}} \left\{ \mu : \lambda \rho _A \otimes \tilde{\rho }_B - \tilde{\rho }_{AB} - \mu \mathbbm {1} \ge 0, ~\textrm{Re} \lbrace {{\,\textrm{Tr}\,}}[X]\rbrace \ge \sqrt{1-\varepsilon ^2}, ~\begin{pmatrix} \rho _{AB} &  X \\ X^\dagger &  \tilde{\rho }_{AB} \end{pmatrix} \ge 0 \right\} . \end{aligned}$$

For the primal problem in the standard form $\sup _{Z \ge 0} \left\{ {{\,\textrm{Tr}\,}}[AZ] : \Phi (Z) \le B \right\} ,$ where *A* and *B* are hermitian operators with suitable block sizes so that all matrix products are well defined and $\Phi$ is a hermiticity-preserving superoperater, one finds:

16 $$\begin{aligned}&Z = \begin{pmatrix} \mu &  0 &  0 \\ 0 &  D &  X \\ 0 &  X^\dagger &  \tilde{\rho }_{AB} \end{pmatrix}, ~~~ A = \begin{pmatrix} 1 &  0 &  0 \\ 0 &  0 &  0 \\ 0 &  0 &  0 \end{pmatrix}, ~~~ B = \begin{pmatrix} 0 &  0 &  0 &  0 &  0 \\ 0 &  1 &  0 &  0 &  0 \\ 0 &  0 &  -1 &  0 &  0 \\ 0 &  0 &  0 &  -\sqrt{1-\varepsilon ^2} &  0 \\ 0 &  0 &  0 &  0 &  \begin{bmatrix} \rho _{AB} &  0 \\ 0 &  0 \end{bmatrix} \\ \end{pmatrix} \end{aligned}$$

17 $$\begin{aligned}&\Phi (Z) = \begin{pmatrix} -(\lambda \rho _A \otimes \tilde{\rho }_B - \tilde{\rho }_{AB} - \mu \mathbbm {1}) &  0 &  0 &  0 &  0\\ 0 &  {{\,\textrm{Tr}\,}}[\tilde{\rho }_{AB}] &  0 &  0 &  0 \\ 0 &  0 &  -{{\,\textrm{Tr}\,}}[\tilde{\rho }_{AB}] &  0 &  0 \\ 0 &  0 &  0 &  -Re\lbrace {{\,\textrm{Tr}\,}}[X]\rbrace &  0\\ 0 &  0 &  0 &  0 &  \begin{bmatrix} 0 &  -X \\ -X^\dagger &  -\tilde{\rho }_{AB} \end{bmatrix} \\ \end{pmatrix}. \end{aligned}$$

We use this to derive the dual problem and show strong duality. The dual problem is $\inf _{Y \ge 0} \left\{ {{\,\textrm{Tr}\,}}[BY]: \Phi ^\dagger (Y) \ge A \right\}$, where ${{\,\textrm{Tr}\,}}[\Phi (Z)Y] = {{\,\textrm{Tr}\,}}[Z\Phi ^\dagger (Y)]$ defines the adjoint superoperator. To derive $\Phi ^\dagger$, set

18 $$\begin{aligned} Y = \begin{pmatrix} W &  0 &  0 &  0 &  0 \\ 0 &  \mu _1 &  0 &  0 &  0 \\ 0 &  0 &  \mu _2 &  0 &  0 \\ 0 &  0 &  0 &  \nu &  0 \\ 0 &  0 &  0 &  0 &  \begin{bmatrix} D_1 &  V \\ V^\dagger &  D_2 \end{bmatrix} \\ \end{pmatrix}, \end{aligned}$$

with $W \in L_+(\mathcal {H}), D_1, D_2, V \in L(\mathcal {H})$ such that $Y \ge 0$ and ${{\,\textrm{Tr}\,}}[BY]$ is well-defined. We then have

19 $$\begin{aligned} {{\,\textrm{Tr}\,}}[\Phi (Z)Y]&= {{\,\textrm{Tr}\,}}\left[ -(\lambda \rho _A \otimes \tilde{\rho }_B - \tilde{\rho }_{AB} - \mu \mathbbm {1})W \right] + {{\,\textrm{Tr}\,}}[\tilde{\rho }_{AB}](\mu _1-\mu _2) - \textrm{Re}\lbrace {{\,\textrm{Tr}\,}}[X] \rbrace \nu \nonumber \\&~~~ - {{\,\textrm{Tr}\,}}\left[ \begin{pmatrix} 0 &  X \\ X^\dagger &  \tilde{\rho }_{AB} \end{pmatrix} \begin{pmatrix} D_1 &  V \\ V^\dagger &  D_2 \end{pmatrix} \right] \end{aligned}$$

20 $$\begin{aligned}&= \mu {{\,\textrm{Tr}\,}}[W] - {{\,\textrm{Tr}\,}}[(\lambda \rho _A \otimes \tilde{\rho }_B-\tilde{\rho }_{AB})W] + {{\,\textrm{Tr}\,}}[(\mu _1 - \mu _2)\tilde{\rho }_{AB}] -\textrm{Re}\lbrace {{\,\textrm{Tr}\,}}[X]\rbrace \nu \nonumber \\&~~~ - {{\,\textrm{Tr}\,}}[XV^\dagger + X^\dagger V + \tilde{\rho }_{AB}D_2] \end{aligned}$$

21 $$\begin{aligned}&= \mu {{\,\textrm{Tr}\,}}[W] + {{\,\textrm{Tr}\,}}[\left\{ (\mu _1-\mu _2)\mathbbm {1} - D_2 - (\lambda M - W) \right\} \tilde{\rho }_{AB}] - \textrm{Re} \lbrace {{\,\textrm{Tr}\,}}[X(\nu \mathbbm {1} + 2V^\dagger )]\rbrace . \end{aligned}$$

In the last equation, we have defined the operator *M*, using an ancillary space $A'$ of the same size as *A*:

22 $$\begin{aligned} M := \mathbbm {1}_A \otimes \lbrace {{\,\textrm{Tr}\,}}_{A'}[W_{A'B}(\rho _{A'} \otimes \mathbbm {1}_B)] \rbrace \implies {{\,\textrm{Tr}\,}}_{AB}[M \tilde{\rho }_{AB}] = {{\,\textrm{Tr}\,}}_{A'B}[ (\rho _{A'}\otimes \tilde{\rho }_B) W]. \end{aligned}$$

This implies the following three relations:

1. 23 $$\begin{aligned} {{\,\textrm{Tr}\,}}[\Phi (Z)Y]&= \mu {{\,\textrm{Tr}\,}}[W] \nonumber \\&\quad + {{\,\textrm{Tr}\,}}\left[ \begin{pmatrix} 0 &  \frac{\nu }{2}\mathbbm {1} +V \\ \frac{\nu }{2}\mathbbm {1} +V^\dagger &  (\mu _1-\mu _2)\mathbbm {1} - D_2 -(\lambda M-W) \end{pmatrix} \begin{pmatrix} D &  X \\ X^\dagger &  \tilde{\rho }_{AB} \end{pmatrix} \right] \end{aligned}$$
  24 $$\begin{aligned}&\implies \Phi ^{\dagger }(Y) = \begin{pmatrix} {{\,\textrm{Tr}\,}}[W] &  0 \\ 0 &  \begin{bmatrix} 0 &  \frac{\nu }{2}\mathbbm {1} +V \\ \frac{\nu }{2}\mathbbm {1} +V^\dagger &  (\mu _1-\mu _2)\mathbbm {1} - D_2 -(\lambda M-W) \end{bmatrix} \end{pmatrix} \end{aligned}$$
2. 25 $$\begin{aligned} \Phi ^\dagger (Y)&\ge A \Leftrightarrow {{\,\textrm{Tr}\,}}[W] \ge 1, \begin{pmatrix} 0 &  \frac{\nu }{2} \mathbbm {1} + V \\ \frac{\nu }{2} \mathbbm {1} + V^\dagger &  (\mu _1 - \mu _2)\mathbbm {1} - D_2 - (\lambda M - W) \end{pmatrix} \ge 0 \end{aligned}$$
3. 26 $$\begin{aligned} {{\,\textrm{Tr}\,}}[BY]&= \mu _1 - \mu _2 - \nu \sqrt{1-\varepsilon ^2} + {{\,\textrm{Tr}\,}}[\rho D_1]. \end{aligned}$$

Hence, the dual program can be written as follows:

27 $$\begin{aligned}&\inf _{\begin{array}{c} W \ge 0\\ \mu _1, \mu _2, \nu \ge 0 \end{array}} \bigg \{ \mu _1 - \mu _2 - \nu \sqrt{1-\varepsilon ^2} + {{\,\textrm{Tr}\,}}[\rho D_1]: {{\,\textrm{Tr}\,}}[W] \ge 1, \nonumber \\&\quad \begin{pmatrix} 0 &  \frac{\nu }{2} \mathbbm {1} + V \\ \frac{\nu }{2} \mathbbm {1} + V^\dagger &  (\mu _1 - \mu _2)\mathbbm {1} - D_2 - (\lambda M - W) \end{pmatrix} \ge 0, ~ \begin{pmatrix} D_1 &  V \\ V^\dagger &  D_2 \end{pmatrix} \ge 0 \bigg \}. \end{aligned}$$

We can simplify the program by defining $\eta :=(\mu _1-\mu _2) \in \mathbbm {R}$ and setting $\nu /2 \rightarrow \nu \ge 0$ in (27), yielding

28 $$\begin{aligned}&\begin{pmatrix} 0 &  \nu \mathbbm {1} + V \\ \nu \mathbbm {1} + V^\dagger &  (\eta \mathbbm {1} - D_2 - (\lambda M - W) \end{pmatrix} \ge 0, ~\begin{pmatrix} D_1 &  V \\ V^\dagger &  D_2 \end{pmatrix} \ge 0. \end{aligned}$$

We can simplify this further by noting that the first condition requires $V = -\nu \mathbbm {1}$, which implies $D_2 \le \eta \mathbbm {1}- (\lambda M -W)$. Setting $D_2=\eta \mathbbm {1}-(\lambda M -W)$ and $V = -\nu \mathbbm {1}$, one finds with the second constraint

29 $$\begin{aligned} \begin{pmatrix} D_1 &  \nu \mathbbm {1}\\ \nu \mathbbm {1}&  \eta -(\lambda M -W) \end{pmatrix}\ge 0, \end{aligned}$$

which holds if and only if (28) holds. For invertible $D_1$ or $\nu =0$, this is implied by the Schur complement, and in case $D_1$ is singular and $\nu >0$, neither (28) nor (29) can be fulfilled. Hence, we can write the final program using (29) and renaming $D_1 \rightarrow D$ as:

30 $$\begin{aligned} \inf _{\begin{array}{c} W, D \ge 0\\ \eta \in \mathbbm {R}, \nu \ge 0 \end{array}} \left\{ \eta - 2\nu \sqrt{1-\varepsilon ^2} + {{\,\textrm{Tr}\,}}[\rho D]: ~ {{\,\textrm{Tr}\,}}[W] \ge 1, ~ \begin{pmatrix} D &  \nu \mathbbm {1} \\ \nu \mathbbm {1} &  \eta \mathbbm {1}- (\lambda M -W)\end{pmatrix} \ge 0 \right\} . \end{aligned}$$

Finally, we conclude strong duality for the primal and dual problem. Set $\tilde{\rho }_{AB} = \rho _{AB}$, $\mu$ such that $\lambda \rho _A \otimes \rho _B - \rho _{AB} - \mu \mathbbm {1} \ge 0$, $X = \sqrt{1-\varepsilon ^2}$, implying $$\begin{pmatrix} \rho _{AB}&X X^\dagger&\tilde{\rho }_{AB} \end{pmatrix} \ge 0$$ and thus being a feasible point of the primal SDP. Let $\alpha> 1,~\nu>0,~\beta >0, ~\eta = \beta + \nu + (\lambda -1)\alpha , ~D=(\nu +\beta ) \mathbbm {1}$ and $W = \alpha \mathbbm {1}$. Then, ${{\,\textrm{Tr}\,}}[W]> 1,~ W> 0,~D>0$ and by (22) $M = \alpha \mathbbm {1}$ and $$\begin{pmatrix} D&\nu \mathbbm {1} \nu \mathbbm {1}&\eta \mathbbm {1} - (\lambda M - W) \end{pmatrix} = \begin{pmatrix} (\nu +\beta ) \mathbbm {1}&\nu \mathbbm {1} \nu \mathbbm {1}&(\nu +\beta ) \mathbbm {1} \end{pmatrix} > 0$$. Consequently, this is a strictly feasible point and strong duality is implied by Slater’s condition (cf. [17]). $\square$

### Proposition 2

If for $\rho _{AB}$ and all $\tilde{\rho }_{AB} \in B^{\varepsilon }(\rho _{AB})$, $\rho _A\otimes \tilde{\rho }_B$ is positive definite, the procedure above converges to $\lambda _{min}$ of (9).

### Proof

Let $\lambda ^i$ be as in (10). Assume that $\lambda ^i > \lambda _{min}$ as in (9). Then, there exists a $\hat{\rho }_{AB}$ and $\hat{\lambda } = \lambda ^i - \hat{\delta }$ with $\hat{\delta } >0$ and $\hat{\lambda }\rho _A \otimes \hat{\rho }_B - \hat{\rho }_{AB} \ge 0$. This implies $\lambda ^i \rho _A \otimes \hat{\rho }_B - \hat{\rho }_{AB} - \hat{\delta }\rho _A \otimes \hat{\rho }_B \ge 0$ and consequently, because $\hat{\delta }> 0$ and by assumption $\rho _A \otimes \hat{\rho }_B > 0$, we must have $\lambda ^i \rho _A \otimes \hat{\rho }_B - \hat{\rho }_{AB} >0$. This indicates that there exist $\hat{\rho }_{AB} \in B^{\varepsilon }(\rho _{AB})$ and $\hat{\mu }>0$ such that $\lambda ^i \rho _A \otimes \hat{\rho }_B - \hat{\rho }_{AB} - \hat{\mu }\mathbbm {1}\ge 0$. This implies that the SDP (11) has a feasible solution $(\mu ^i, \rho ^{i+1}_{AB})$ and consequently $\lambda ^i \rho _A \otimes \rho ^{i+1}_B - \rho ^{i+1}_{AB} > 0$. Thus, using $\rho ^{i+1}_{AB}$ for the next iteration in (10), will return a $\lambda ^{i+1} < \lambda ^i$. In summary, we have shown that if $\lambda ^i > \lambda ^{min}$, then there exists a feasible solution $\mu ^i$ of the SDP (11), provided that all states have full rank. This solution implies a feasible improved solution $\lambda ^{i+1}<\lambda ^i$ of (10) for the next iteration. Due to the strong duality of the SDP involved (see Proposition 1), if it exists, a feasible solution will be found by the program. Failing to do so in step 3 in Algorithm 1 consequently implies that no feasible $\mu ^i > 0$ exists and therefore $\lambda ^i = \lambda _{min}$. Proposition 2.3. in [14] implies that this seesaw/mountain-climbing-type algorithm converges to the optimal solution after a finite number of steps. $\square$

Before concluding, we briefly discuss the role of the rank constraint $\rho _A \otimes \hat{\rho }_B > 0$: If this constraint is violated, then $\lambda ^i > \lambda _{min}$ does not necessarily imply $\lambda ^i \rho _A \otimes \hat{\rho }_B - \hat{\rho }_{AB} >0$. Instead, $\lambda ^i \rho _A \otimes \hat{\rho }_B - \hat{\rho }_{AB}$ and $\rho _A \otimes \hat{\rho }_B$ may have nontrivial intersecting kernels, such that $\lambda ^i \rho _A \otimes \hat{\rho }_B - \hat{\rho }_{AB} - \hat{\delta }\rho _A \otimes \hat{\rho }_B \ge 0$ holds for some $\hat{\delta }>0$, but $\lambda ^i \rho _A \otimes \hat{\rho }_B - \hat{\rho }_{AB} - \mu \mathbbm {1}< 0$ for all $\mu > 0$. In this case, the SDP in (11) results in $\mu =0$ and the algorithm stops without necessarily having found the optimal solution $\lambda _{min}$.

## Conclusion

In this work, we developed an SDP-based iterative algorithm to compute the smooth max-mutual information $I^\varepsilon _{\max }(\rho _{AB})$ of bipartite quantum states. The algorithm was proven to be accurate under the assumption that for all states $\tilde{\rho }_{AB}$ in the smoothing environment around $\rho _{AB}$, the marginal states $\rho _A \otimes \tilde{\rho }_B$ have full rank.

A key contribution of this work is the characterization of a previously unexplored semidefinite program, which forms a core component of the iterative procedure. We established its primal and dual formulations and proved strong duality, thereby laying a rigorous foundation for its use in quantum information theory. Our results extend the landscape of SDP-based techniques for evaluating operational quantities, complementing existing methods as used for the computation of, e.g., the hypothesis testing mutual information [12, 18], or the conditional smooth min- and max-entropy [13]. Notably, since the smooth max-mutual information can be combined with the hypothesis testing mutual information to lower-bound the one-shot distillable key (cf. Ref.  [2]), our algorithm offers a tool for estimating cryptographic capacities of quantum states that satisfy the rank constraints.

If the constraint concerning the rank of marginal states in the smoothing environment $B^\varepsilon (\rho _{AB})$ is not fulfilled, the algorithm may not yield optimal solutions. In the worst case, the algorithm stops in the first iteration, returning the (non-smoothed) max-mutual information $I_{max}(\rho _{AB}):= D_{max}(\rho _{AB}||\rho _A \otimes \rho _B)$. Hence, $I_{max}(\rho _{AB})$ provides an upper bound for the results of the algorithm. However, the algorithm terminates prematurely only if the state $\rho _{AB}$ itself or the exact maximizer states $\tilde{\rho }_{AB}$ in the smoothing environment violate the constraint. Otherwise, the algorithm still converges, even if the environment contains (non-maximizing) states that violate the constraint.

Future work may focus on relaxing the rank assumptions, which would broaden the applicability of our method to general quantum states. In Ref. [19], it is shown that the smooth max-mutual information of $\rho _{AB}$ is invariant under local unitary transformations $U_A$ acting on *A* and general local isometric transformations $V_{B' \rightarrow B}$, mapping a smaller system $B'$ to *B*. If for a state $\rho _{AB}$ the rank condition is violated, but there exists a state $\hat{\rho }_{AB'}$ with $\rho _{AB} = U_A\otimes V_{B' \rightarrow B}~\hat{\rho }_{AB'}~U_A^\dagger \otimes V_{B' \rightarrow B}^\dagger$ that satisfies the condition, then the smooth max-mutual information can be computed with the presented algorithm for $\hat{\rho }_{AB'}$, instead. One potential way to lift the rank conditions is to analyze for which states such a pre-image under local transformations exists. A systematic empirical study of convergence rates and required iteration counts, as well as exploring the significance of the rank constraint, would provide a complementary numerical investigation to the analytical scope of this work. Moreover, the fully characterized SDP presented in Proposition 1 may find utility beyond this context, potentially serving as a building block for evaluating other information-theoretic quantities or optimization problems in quantum theory and related fields.

## Data Availability

No datasets were generated or analyzed during the current study.
